# Supplementary material for: Cannabidiol and Cannabigerol Inhibit Cholangiocarcinoma Growth In Vitro via Divergent Cell Death Pathways
Source: Biomolecules. 2022 Jun 20;12(6):854. doi: 10.3390/biom12060854 (PMC9221388; doi:10.3390/biom12060854)
Supplement: Supplementary file 1 [file biomolecules-12-00854-s001.zip › biomolecules-1777088-supplementary.pdf]

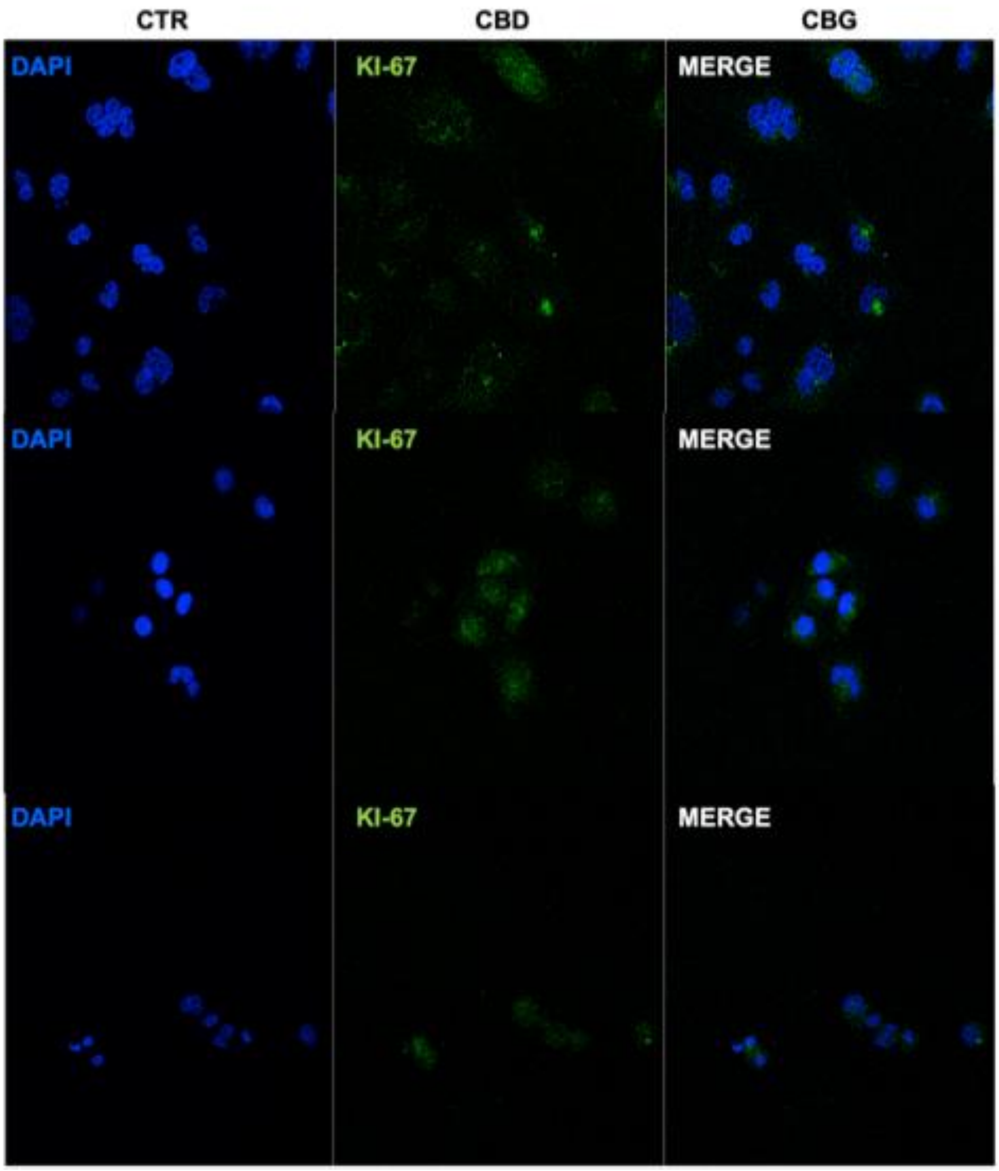


**Figure S1.** DAPI staining of nucleus (blue) and Alexfluor488 staining of KI-67 (green) for control (0.1% DMSO), 100 μM CBD and 100 μM CBG at 24 hours post treatment. DAPI staining shows cells actively dividing in control, with several nuclei appear to be dividing; CBD and CBG show low cell density with no bi-lobed nuclei.

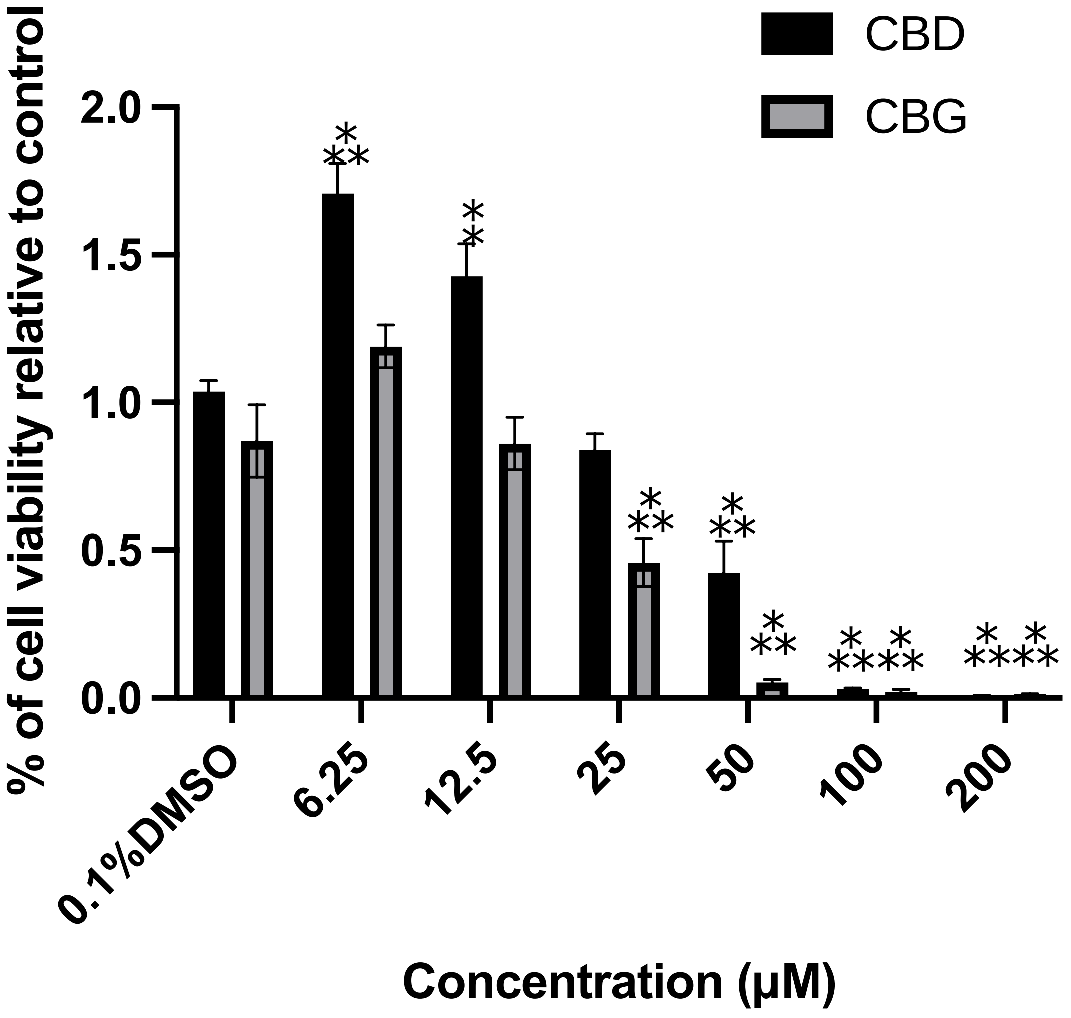


**Figure S2.** MTT assay results showing cell proliferation in H69 48-hours post treatment with control (0.1% DMSO) CBD and CBG. *$P < 0.05$, ** $P < 0.01$, *** $P < 0.001$ v.s. control.

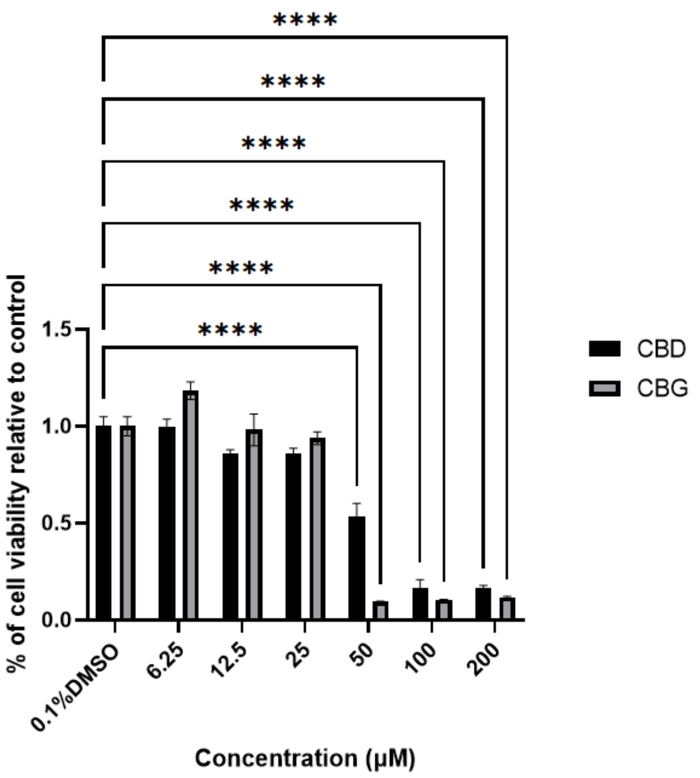


(A)

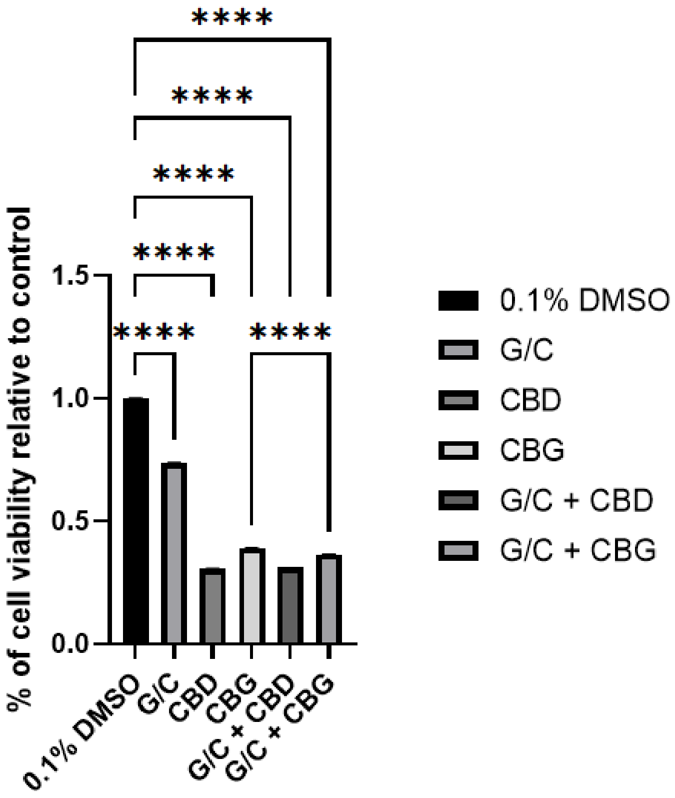


(B)

**Figure S3.** CBD and CBG reduced proliferation in MzChA-1 cells; CBG synergizes with Gemcitabine/Cisplatin (G/C) treatment. **(A)** MTT assay results showing cell viability in Mz-ChA-1 48-hours post treatment with control (0.1% DMSO), CBD or CBG. **(B)** MTT assay results showing cell viability in HuCC-T1 24 hours post treatment with G/C (100 μM), CBD (100 μM), CBG (100 μM), G/C (100 μM) + CBD (100 μM), and G/C (100 μM) + CBG (100 μM). **** $P < 0.0001$.

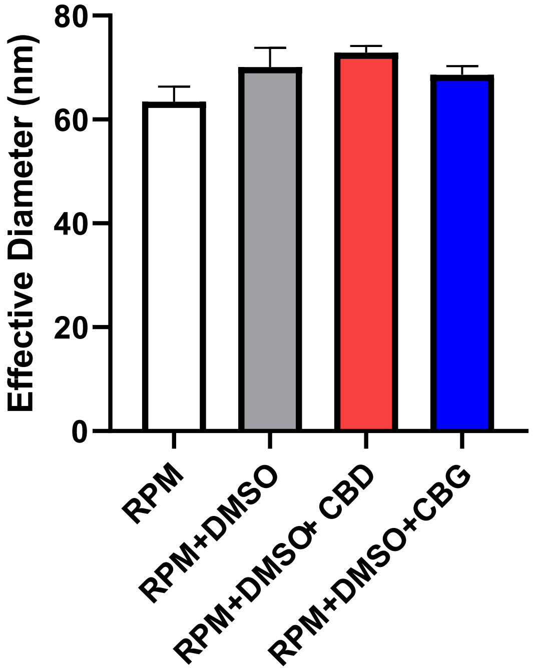


(A)

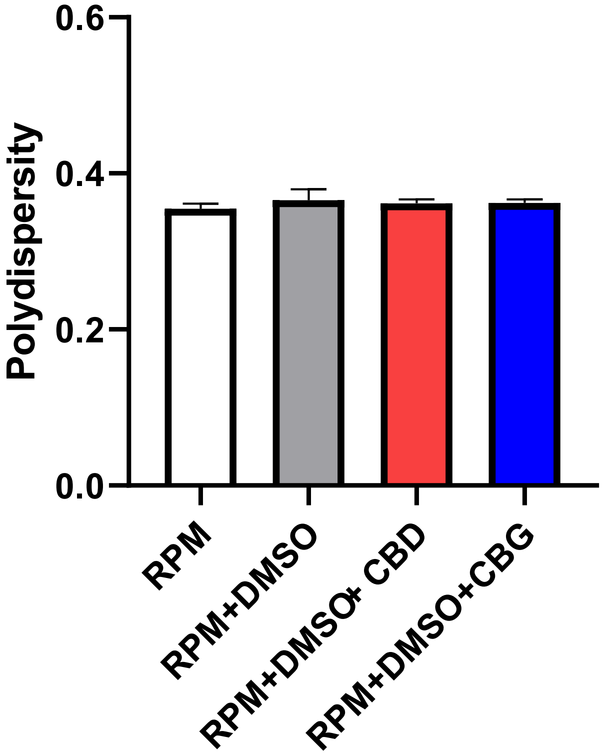


(B)

**Figure S4.** CBD and CBG did not change the effective diameter or the polydispersity. The effective diameter **(A)** and the polydispersity **(B)** is measured in four different groups in triplicates. The four groups are RPM: the blank culture medium; RPM+DMSO: the culture medium with DMSO; RPM+DMSO+CBD: culture medium with DMSO and CBD (100 μM), RPM+DMSO+SBD: culture medium with DMSO and CBG (100 μM).
